# Cryo-EM structure of the human concentrative nucleoside transporter CNT3

**DOI:** 10.1371/journal.pbio.3000790

**Published:** 2020-08-10

**Authors:** Yanxia Zhou, Lianghuan Liao, Chen Wang, Jialu Li, Pengliang Chi, Qingjie Xiao, Qingting Liu, Li Guo, Linfeng Sun, Dong Deng

**Affiliations:** 1 Division of Obstetrics, Key Laboratory of Birth Defects and Related Disease of Women and Children of MOE, State Key Laboratory of Biotherapy, West China Second Hospital, Sichuan University, Chengdu, China; 2 Hefei National Laboratory for Physical Sciences at Microscale, CAS Centre for Excellence in Molecular Cell Science, School of Life Sciences, University of Science and Technology of China, Hefei, China; University of Zurich, SWITZERLAND

## Abstract

Concentrative nucleoside transporters (CNTs), members of the solute carrier (SLC) 28 transporter family, facilitate the salvage of nucleosides and therapeutic nucleoside derivatives across the plasma membrane. Despite decades of investigation, the structures of human CNTs remain unknown. We determined the cryogenic electron microscopy (cryo-EM) structure of human CNT (hCNT) 3 at an overall resolution of 3.6 Å. As with its bacterial homologs, hCNT3 presents a trimeric architecture with additional N-terminal transmembrane helices to stabilize the conserved central domains. The conserved binding sites for the substrate and sodium ions unravel the selective nucleoside transport and distinct coupling mechanism. Structural comparison of hCNT3 with bacterial homologs indicates that hCNT3 is stabilized in an inward-facing conformation. This study provides the molecular determinants for the transport mechanism of hCNTs and potentially facilitates the design of nucleoside drugs.

## Introduction

Nucleosides play crucial roles in cell homeostasis, functioning as nucleotide precursors [1] and signaling molecules [2]. In mammals, nucleosides can be synthesized de novo or via a salvage pathway. However, many human cells are unable to synthesize nucleosides via the de novo biosynthetic pathway [3]. Therefore, to achieve nucleoside homeostasis, the nucleoside transporter facilitated nucleoside salvage pathway is of great importance [4]. In addition, nucleoside transporters on the plasma membrane have been implicated in the cellular uptake of several therapeutic nucleoside derivatives that are used to treat viral infections and cancers [3,5,6].

Solute carrier (SLC) transporters facilitate the transport of multiple substrates and play important roles in physiological processes [7]. Structural investigations are vital for understanding the transport mechanism of SLC transporters [8–10]. To date, several nucleoside transporters in mammals have been identified and classified into two SLC transporter families: concentrative nucleoside transporters (CNTs, the SLC28 family) and equilibrative nucleoside transporters (ENTs, the SLC29 family) [4,11]. ENTs are restricted to eukaryotes, whereas CNTs are also found in prokaryotes. Recently, the crystal structure of the well-characterized human ENT1 was determined and provides mechanistic insights into its inhibition by adenosine reuptake inhibitors [12]. Although CNTs have been biochemically characterized over the past 2 decades, the molecular mechanism of nucleoside transport by CNTs remains unknown [13–17]. Structural investigations of bacterial CNT homologs, vcCNT [18,19] and CNTnw [20], have suggested that these two CNTs are good models for understanding the transport mechanism of human CNTs and have provided information about the structural fold of the core domain of CNTs, the nucleoside specificity of CNTs, and the elevator-like movement of the alternating access mechanism [21,22].

Human CNT (hCNT) 3 (SLC28A3), one of the three members of the SLC28 family, is classified as a sodium–nucleoside or proton–nucleoside symporter [23,24]. Compared with the other two members, hCNT3 has the broadest distribution, substrate specificity, and transport activity for nucleoside-derived drugs [25]. hCNT3 is also an important mediator of drug response and resistance [26]. Previous studies have shown that the truncated isoform (Δ1–69) of hCNT3, named CNT3ins, is an endoplasmic reticulum membrane protein with nucleoside transport activity [27]. Here, we report the cryo-EM structure of CNT3ins at an overall resolution of 3.6 Å. Structural comparison between CNT3ins and bacterial homologs together with nucleoside binding assays unveiled the substrate selectivity and alternating access transport mechanism of hCNTs.

## Results

### Characterization of full-length hCNT3 and CNT3ins

We purified full-length wild-type hCNT3 and CNT3ins with monodispersed peaks in detergent micelles (S1 Fig). Both proteins were eluted from size-exclusion chromatography at approximately 14 mL, which suggested that hCNT3 and CNT3ins present identical oligomerization states. Considering the trimeric architecture of bacterial CNTs [18] and the cross-linking assay of hCNT3 [28], we speculated that purified hCNT3 and CNT3ins existed as trimers in detergent micelles.

**Fig 1:**
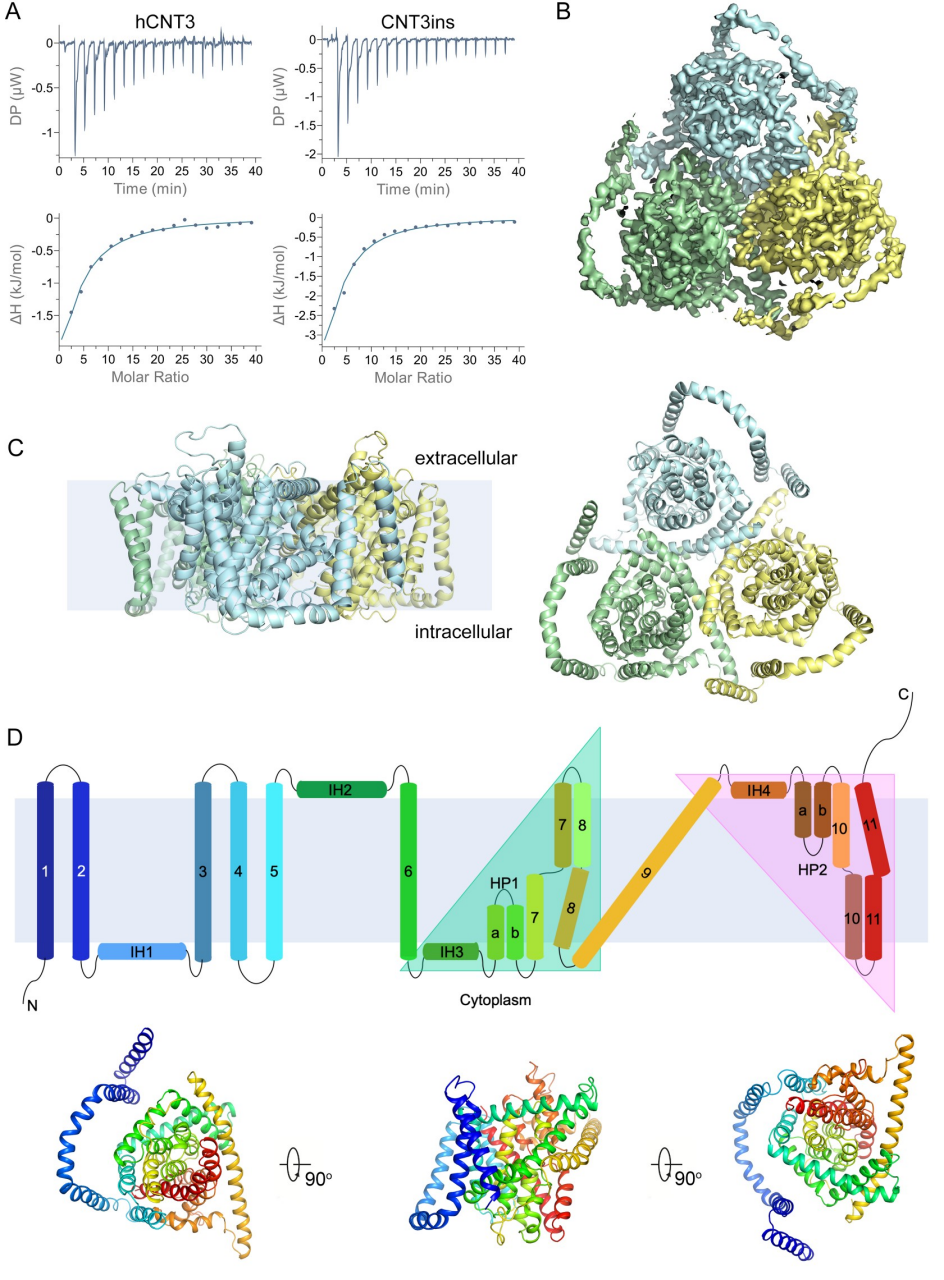
Characterization and structure of CNT3ins. (A) The binding affinities between hCNT3/CNT3ins and uridine were measured by ITC. The underlying data for this figure can be found in S1 Data. (B) The density of trimeric CNT3ins is shown, and the protomers are colored pale cyan, pale green, and pale yellow. (C) CNT3ins is shown in cartoon representation, and the protomers are colored pale cyan, pale green, and pale yellow. (D) Topology and structure of monomeric CNT3ins. (Top) Topology diagram with helices represented by rainbow-colored cylinders. Inverted repeat regions in the core domain of hCNT3 are covered by triangles. (Bottom) Structure of monomeric hCNT3 with helices colored to correspond to equivalent helices in the topology diagram. CNT, concentrative nucleoside transporter; hCNT, human CNT; DP, differential power; ΔH, the enthalpy of binding; HP, hairpin-shaped helix; IH, interfacial helix; ITC, isothermal titration calorimetry.

To further validate the functions of hCNT3 and CNT3ins, isothermal titration calorimetry (ITC) was carried out. As the results show, both hCNT3 and CNT3ins proteins bind uridine with equal binding affinity (approximately 325 μM for hCNT3 and approximately 368 μM for CNT3ins), which indicates that CNT3ins share similar uridine-transporting activity (Fig 1A). The uridine binding affinity of hCNT3 was also detected under sodium-free condition. Interestingly, there was no binding detected between uridine and hCNT3 under this condition (S2A Fig). This observation is consistent with previous speculation that sodium binding probably forms the accurate nucleoside binding pocket of CNTs and facilitates nucleoside binding [29]. Furthermore, the binding of cytidine and thymidine was detected via ITC. The binding affinity of cytidine and thymidine reached approximately 260 μM and approximately 603 μM, respectively (S2B and S2C Fig).

### The overall structure of CNT3ins

Considering that oligomeric hCNT3 has a molecular weight of greater than 200 kDa, we tried to solve the structure of hCNT3 via single-particle cryogenic electron microscopy (cryo-EM). We first focused on the full-length hCNT3 in different detergents, including n-dodecyl-β-D-maltoside (DDM) and lauryl maltose neopentyl glycol (LMNG). However, the preliminary image collections did not present reasonable homogeneity for further structural determination. Then, we tried to purify CNT3ins in 0.1% (w/v) digitonin, which has also been used for the structural determination of human γ-secretase [30], and analyzed its structure using single-particle cryo-EM (S3A Fig). Particles from the acquired cryo-EM images displayed good contrast (S3B Fig). Two-dimensional (2D) class averages of the selected particles showed clear features of the overall structure of hCNT3 (S3B Fig). The final three-dimensional (3D) density map was reconstructed from 99,489 particles with C3 symmetry, reaching an overall resolution of 3.6 Å (Fig 1B, S3C and S3D Fig, S1 Table). The local resolution of the core domain was approximately 3 Å, which allowed us to build side chains with a high degree of accuracy (S3E and S4 Figs). The processing scheme for structural determination is summarized in S5 Fig. CNT3ins consists of three protomers (Fig 1B), a trimeric shamrock-shaped architecture that is formed through trimeric contacts of the central helices from each protomer (Fig 1C). Previous structural investigations have revealed the trimeric architecture of bacterial CNTs [18], which is also consistent with previous biochemical studies of hCNT3 [28].

### The monomeric structure of CNT3ins

In the structure of CNT3ins, density is clearly resolved for residues 100–614 of each protomer (Fig 1B), which can be divided into the core domain and additional N-terminal transmembrane helices (TMs). Each protomer of hCNT3 is formed by 11 TMs (TM1–11), which is consistent with the previously predicted 11 TM topology of hCNTs. However, the additional N-terminal helices are linked by an interfacial helix (IH1) between TM2 and TM3 (Fig 1D). This N-terminal region (TM1–3 and IH1) of hCNT3 shares high sequence homology with hCNT1 and hCNT2 (S6 Fig), which suggests that the N-terminal helices are a common feature of hCNTs. The core domain of hCNT3 shares a similar topology with its bacterial homologs, vcCNT and CNTnw, containing eight TMs (TM4–11), three IHs (IH2–4), and a pair of hairpin-shaped helices (HPs) with reverse orientations (HP1 and HP2) (Fig 1D and S6 Fig).

The core domain of hCNT3 is composed of two subdomains, including the scaffold domain (TM4, TM5, TM6, TM9, and IH2) and the transport domain (IH3, HP1, TM7, TM8, IH4, HP2, TM10, and TM11). TM4 and TM5 are located in the periphery of the core domain. IH2, TM6, and TM9 mediate the trimerization contacts between the protomers of hCNT3. The transporter domain has two structurally inverted repeats linked by TM9 (Fig 1D). Each repeat contains an IH, a helical hairpin, an unwound helix, and another TM (Fig 1D). Previous studies have identified the important roles of the helical hairpins and unwound helices of transporters in substrate recognition and alternating access [21,22]. ConSurf evolutionary conservation analysis also revealed the high sequence homology and functional relevance of the core domains of the CNTs (S7 Fig).

### Putative substrate and sodium binding sites of hCNT3

**Fig 2:**
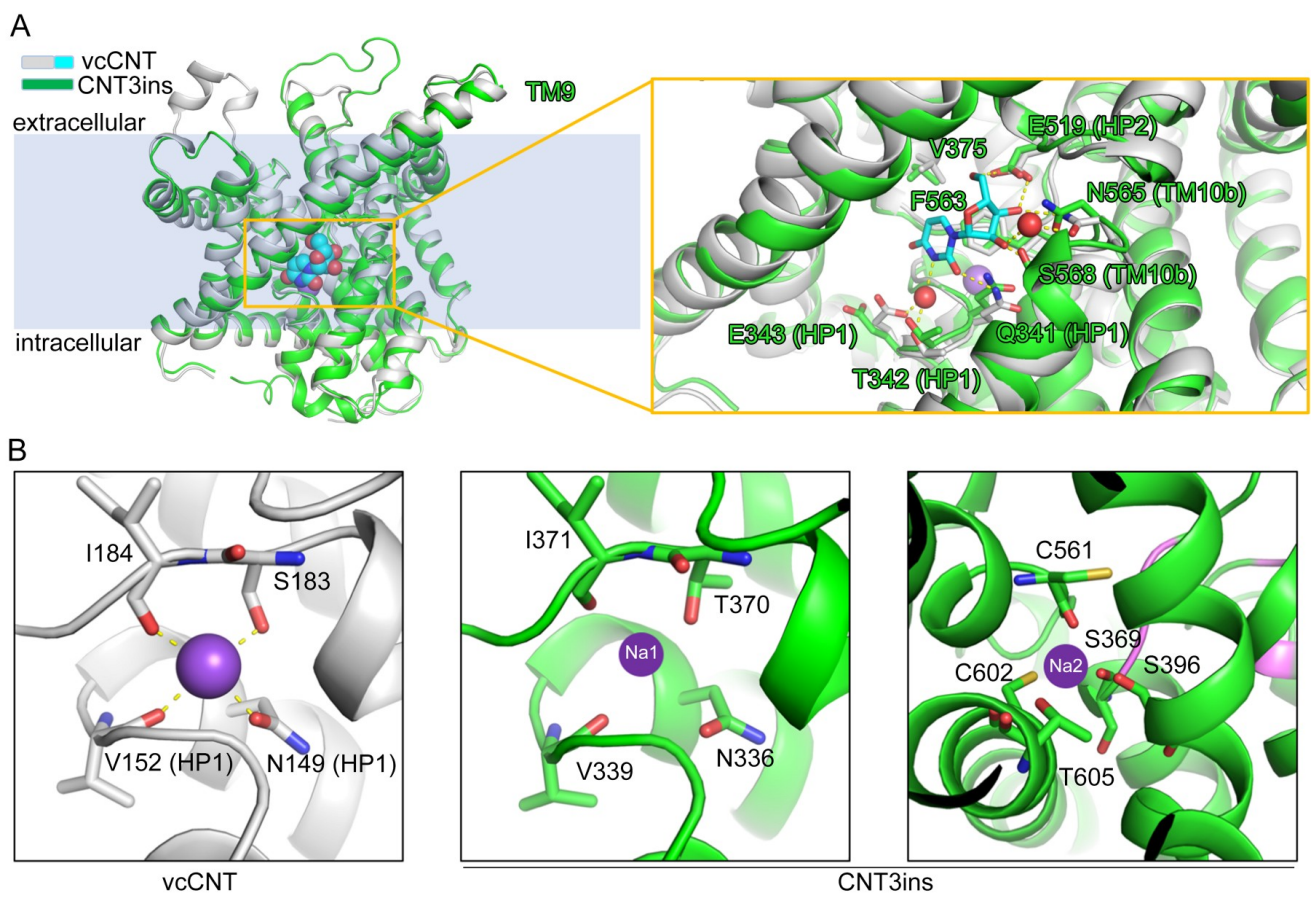
Putative substrate binding site and sodium binding sites of CNT3ins. (A) Superposition of the core domains of CNT3ins and vcCNT (PDB code: 3TIJ). CNT3ins and vcCNT are shown as ribbons and colored green and white, respectively. Uridine is shown as a sphere and is colored cyan. In the right panel, the coordinate residues are shown as sticks. Hydrogen bonds are shown as yellow dashed lines. Conserved residues of hCNT3 are labeled. (B) The sodium binding site of vcCNT is shown in the left panel. Sodium is shown as a purple sphere, and hydrogen bonds are shown as yellow dashed lines. Two putative sodium binding sites of hCNT3 are shown in the middle and right panel. The coordinate residues are highly conserved. CNT, concentrative nucleoside transporter; hCNT, human CNT; HP, hairpin-shaped helix; PDB, Protein Data Bank; TM, transmembrane helix.

Considering that the structure of CNT3ins was obtained without nucleoside and no sodium ions were observed in the structure of CNT3ins, we used the structural superposition to identify the putative nucleoside and sodium binding sites of hCNT3. The superposition of core domains, scaffold domains, and transport domains from CNT3ins and bacterial vcCNT presented nearly identical conformations, with root-mean-square deviations (RMSDs) of 1.1 Å over 368 Cα atoms, 1.3 Å over 138 Cα atoms, and 1.0 Å over 222 Cα atoms (S8 Fig). In the structure of vcCNT, the nucleoside is stabilized in the pocket located in the middle of the transport domain (Fig 2A). The substrate binding site of vcCNT consists of residues from HP1, HP2, TM4, and TM7 (Fig 2A). As superposition shows, the residues involved in nucleoside binding of hCNT3 (Q341, T342, E343, V375, F563, E519, N565, and S568) and vcCNT are identical, which indicates that hCNT3 possesses similar nucleoside selectivity to vcCNT (Fig 2A and S6 Fig).

Because a nucleoside consists of a ribose and a nucleobase, the interactions between the nucleoside and residues of hCNT3 involve two aspects. First, the charged or polar residues (E519, N565, and S568) from HP2 and TM10, which are invariant among the hCNTs, interact with ribose through hydrogen bonds. Second, Q341, T342, and E343 from HP1 and V375 from TM7 are responsible for interacting with the nucleobase. Moreover, it was observed that F563 plays an important role in substrate recognition by CH-π and π-π interactions (Fig 2A). To validate the importance of the polar or charged residues for nucleoside recognition, the uridine binding of hCNT3 mutants was measured via ITC, including E343A, E519Q, E519A, N565A, and S568P. E519Q, E519A, and S568P showed a dramatic decrease in uridine binding (no binding). The uridine binding affinity of hCNT3_E343A_ was approximately 1.19 mM, whereas N565A maintained uridine binding with dissociation constant (Kd) of approximately 280 μM (S9 Fig).

Interestingly, hCNT3 exhibits a unique sodium/nucleoside cotransport stoichiometry of 2:1, whereas CNT1 and CNT2 have stoichiometries of 1:1 [24]. The first sodium binding site was identified in the structure of vcCNT [18]. In hCNT3, the invariant residues (N336, V339, and I371) and T370 (Ser183 in vcCNT) surround and constitute the sodium binding site (Fig 2B). Because residues for nucleoside binding localize on HP1 and TM10b, we speculate that the role of sodium binding is to form a mature nucleoside binding site. To identify the second sodium binding site, we analyzed a polymorphism of hCNT3, C602R, which has an altered sodium/nucleoside stoichiometry of 1:1 [25,31,32]. This indicates that C602 is part of the second sodium binding site and is located near the first site (Fig 2B). Several residues, including S369, S396, C561, C602, and T605, are required to form the sodium binding cavity (Fig 2B). Despite the different sodium/nucleoside cotransport stoichiometry, the natural polymorphic variant (C602R) of hCNT3 maintains reduced nucleoside transport activity [25,31], indicating the primary role of the first sodium binding site for nucleoside transport. Interestingly, we could not detect uridine binding of CNT3_C602R_ via ITC (S9 Fig). This result indicates that the putative second sodium binding site is also very important for increasing nucleoside binding affinity.

### Inward-facing conformation and alternating access transport mechanism of hCNT3

Previous structural studies of bacterial CNTs have elucidated the conformations of the multiple steps of the alternating access transport cycle, including inward-facing, intermediate, and outward-facing conformations [20], revealing that CNTs transport substrates using an elevator-like motion coupled with state-dependent conformational changes of HP1 [20]. Furthermore, the structures of bacterial CNTnw show that each of the three protomers has different conformations, indicating that each protomer achieves substrate transport independently.

The three protomers of CNT3ins share identical conformations as a result of data processing with C3 symmetry. Therefore, one protomer was selected to determine the state of CNT3ins. The elevator-like motion of the transport domains of bacterial CNTs reveals the unchangeable trimeric helices in the scaffold domain, including IH1, TM3, and TM6 (corresponding to IH2, TM6, and TM9 in hCNT3). During alternating access transport, the movement of the transport domain involves upward/downward translocation that exposes the sodium and nucleoside binding sites above/below TM6 (TM9 in hCNT3) to make these sites accessible to both sides of the plasma membrane. The high sequence conservation and the superposition of the transport domains of CNTs (S6 and S8 Figs) indicated that hCNT3 shares a similar transport process and principle function to bacterial CNTs.

**Fig 3:**
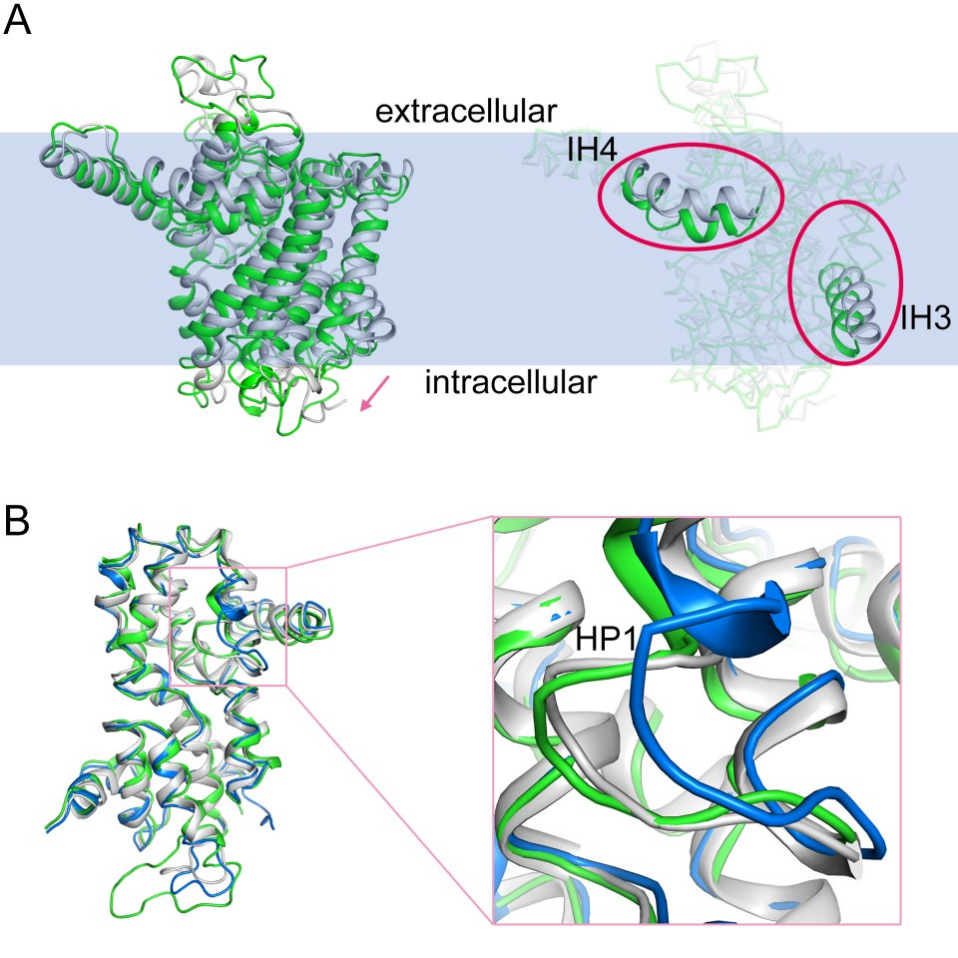
Inward-facing conformation of CNT3ins. (A) Superposition of monomeric CNT3ins (green) and vcCNT (white). To identify the downward motion, the motion is represented by the pink arrow; IH3 and IH4 are highlighted by pink ellipses. To make the figure succinct, the additional helices of hCNT3 are hidden. (B) Superposition of the transport domains of CNT3ins (green), vcCNT (white, inward-facing state), and CNTnw (blue, inward-open state). CNT, concentrative nucleoside transporter; hCNT, human CNT; HP, hairpin-shaped helix; IH, interfacial helix.

To investigate this, we superposed the protomer of hCNT3 with that of the bacterial CNTs to explore the state of the cryo-EM structure of hCNT3 (Fig 3). By considering the upward or downward movement of the transport domain and the unchanged trimeric helices, we superposed the structures of vcCNT and hCNT3 by aligning the central trimeric domain (IH2, TM6, and TM9). Surprisingly, the transport domain of hCNT3 undergoes a slight downward (inward) movement (Fig 3A). Moreover, for IH3 and IH4 in the transport domain, the linkers between the transport domain and scaffold domain moved down accordingly (Fig 3A). The observed position of the transport domain indicates an inward-facing state of hCNT3.

Interestingly, as shown in a previous study, the inward-facing open state of bacterial CNTnw has a nearly identical conformation with the inward-facing state of vcCNT, except for a slight structural change in HP1 [20]. By superposing the transport domains of the protomers of the CNTs, the transport domain can be shown to exhibit a rigid feature (Fig 3B). The dynamic conformations of HP1 could also be observed (Fig 3B). The diversity of conformations of HP1 indicates that the HP1 motif plays an important role in substrate binding and that nucleoside binding can stabilize the conformation of HP1 in all CNTs.

Our cryo-EM structure of hCNT3 was obtained under nucleoside-free conditions similar to those of the crystal structures of CNTnw (Protein Data Bank [PDB] codes: 5L2A, 5L2B, 5L24, 5L27, and 5U9W). However, the conformation of HP1 in hCNT3 is almost identical to that of bacterial CNTs in the substrate-bound inward-facing conformations (PDB codes: 3TIJ and 5L26) and slightly different from that of the CNTs in the substrate-free inward-facing conformations (protomers A and B of the structures of CNTnw mentioned above). This also suggests that hCNT3 was captured in an inward-facing state. The additional slight elevator-like downward motion of the transport domain of hCNT3 is, however, different from that of the substrate-bound inward-facing states of CNTnw and vcCNT.

## Discussion

In this study, we solved the cryo-EM structure of CNT3ins in an inward-facing conformation. Interestingly, the N-terminal motif of hCNT3 determines plasma membrane targeting [33]. CNT3ins lacks the 69 N-terminal residues and is an endoplasmic reticulum membrane protein with nucleoside transport activity [27]. Our results also confirmed the uridine binding capacity of CNT3ins, which suggested the similar nucleoside transport activity of CNT3ins as wild-type hCNT3.

Bacterial homologs of CNTs only contain conserved C-terminal domains (scaffold domain and transport domain) but lack the three N-terminal TMs (TM1–3) (S6 Fig). Given that bacterial CNTs have been identified as sodium–nucleoside or proton–nucleoside symporters, the conserved C-terminal scaffold domain and transport domain are responsible for the nucleoside transport activity of bacterial CNTs [18,34]. Therefore, the N-terminal helices seen in the structure of CNT3ins are not essential for nucleoside transport. However, truncated hCNT1 lacking these helices retained lower activity than wild-type hCNT1 [16]. This indicates that these helices are important for the expression and translocation of CNTs.

hCNT3 was identified as a nucleoside transporter with broad selectivity [35]. The kinetic parameters of uptake of various nucleosides by hCNT3 were quite similar, which indicates a similar binding affinity among the nucleosides, whereas hCNT1 and hCNT2 exhibit specificity for pyrimidine and purine nucleosides, respectively [4]. Previous studies have identified a series of mutations that can convert hCNT1 into an hCNT2-like transporter (S319G/Q320M/S353T/L354V) or an hCNT3-like transporter with broad concentrative nucleoside transport activity (S319G/Q320M) [13]. Sequence alignment between the CNTs revealed the positions of specific residues (G340/Q341/S374/V375) involved in substrate selectivity in hCNT3. All four of these residues are located in the binding pocket and are involved in the recognition of nucleobases, indicating their importance in substrate selectivity. Interestingly, vcCNT with identical residues involving nucleoside binding similar to those of hCNT3 bind purine and pyrimidine with distinguishable binding affinities (approximately 36 μM for uridine, approximately 61 μM for cytidine, and approximately 470 μM for adenosine, respectively) [19]. Therefore, further in vitro binding assays and transport assays of hCNT3 should be carried out.

hCNT3 exhibits a unique sodium/nucleoside cotransport stoichiometry of 2:1 as well as proton/nucleoside cotransport stoichiometry of 1:1. Previous studies have identified many mutations altering sodium/nucleoside cotransport stoichiometry [25,36–38]. However, most of these studies showed a decrease in the transport activity and nucleoside binding [25,36–38]. The putative second sodium binding site presented in a previous study also needs further experimental verification [25]. Considering the proton/nucleoside cotransport capacity of hCNT3 [29], a uridine binding assay in MES/KOH buffer at pH 5.5 was carried out. Surprisingly, a lower uridine binding affinity to hCNT3 was detected under the conditions of pH 5.5 with 500 mM NaCl, and no obvious binding was detected under sodium-free MES/KOH (pH 5.5) buffer conditions (S10 Fig). Probably, purified hCNT3 was stabilized in a specific conformation in detergent micelles. Interestingly, the sodium/melibiose symporter melibiose permease (MelB) also transports melibiose coupling with protons [39]. The proton-coupling mechanism of MelB proposed that the negatively charged residues in a single cation binding site selectively coordinate sodium and protons [39]. Notably, the proton-coupling mechanism of hCNT3 is more complicated because of the two proposed cation binding sites.

In general, this study sheds light on understanding the selective nucleoside binding and distinct coupling transport mechanism of human CNT3. This study also potentially facilitates the design of nucleoside drugs.

## Materials and methods

### Protein expression and purification

Codon-optimized cDNA of human CNT3 was synthesized by GENEWIZ. For structural investigation, the full-length truncated version and mutations of hCNT3 were subcloned into pFastBac1 (Invitrogen) with a C-terminal Strep-tag. Recombinant hCNT3 protein was expressed using the pFastBac baculovirus expression system (Invitrogen). Briefly, bacmid DNA was generated in DH10Bac cells, and the resulting baculoviruses were generated and amplified in Sf9 insect cells (Invitrogen). All hCNT3 proteins were overexpressed in Sf9 insect cells (Invitrogen) and grown in SIM SF medium (Sino Biological Inc.). At 60 h after viral infection, the cells were collected using binding buffer containing 100 mM Tris, 150 mM NaCl, and 1 mM EDTA at pH 8.0.

For structural determination, the cells were solubilized in binding buffer containing protease inhibitors (0.8 mM aprotinin, 2 mM pepstatin, 5 mg/ml leupeptin) and 1% (w/v) digitonin (Sigma Aldrich) at 4°C for 2 h. After high-speed centrifugation at 20,000*g* for 30 min at 4°C, the detergent-soluble fraction was harvested and incubated with StrepTactin Sepharose (GE Healthcare) at 4°C for 1 h. The StrepTactin Sepharose was washed extensively with binding buffer containing 0.1% (w/v) digitonin four times. The protein eluted from StrepTactin Sepharose with wash buffer plus 10 mM d-desthiobiotin. The protein was concentrated and purified by size-exclusion chromatography (Superose 6 10/300; GE Healthcare) in buffer containing 25 mM Tris (pH 8.0), 150 mM NaCl, and 0.1% (w/v) digitonin. The peak fraction was pooled and concentrated to 6 mg/ml for cryo-EM analysis.

For ITC, cells were solubilized in binding buffer (100 mM Tris, 150 mM NaCl, and 1 mM EDTA at pH 8.0) containing protease inhibitors (aprotinin at 0.8 mM, pepstatin at 2 mM, leupeptin at 5 mg/ml) and 2% (w/v) DDM (Anatrace) at 4°C for 2 h. After high-speed centrifugation at 20,000*g* for 30 min at 4°C, the detergent-soluble fraction was incubated with StrepTactin Sepharose (GE Healthcare) at 4°C for 1 h. The resin was washed with binding buffer containing 100 mM Tris, 150 mM NaCl, and 1 mM EDTA at pH 8.0 and 0.02% (w/v) DDM four times. The protein eluted with wash buffer plus 10 mM d-desthiobiotin. The protein was concentrated and purified by size-exclusion chromatography (Superose 6 10/300; GE Healthcare) in buffer containing 10 mM HEPES/KOH (pH 8.0), 100 mM KCl, and 0.02% DDM with or without 500 mM NaCl. The peak fractions were pooled together and concentrated to 50 μM (the molarity of the protomer of CNT3).

### Electron microscopy

Four-microliter aliquots of purified CNT3ins (Δ1–69) in digitonin at a concentration of 6 mg/ml were placed on glow-discharged holey carbon grids (Quantifoil Au R1.2/1.3, 300 mesh) and flash frozen in liquid ethane using the FEI Vitrobot. Images were recorded manually on the FEI Titan Krios electron microscope at 300 kV with a nominal magnification of 22,500×. Defocus values in the data set ranged from −1.5 to −2 μm. A Gatan K2-Summit detector was used in superresolution mode. Each image was acquired with an exposure time of 8 s and dose fractionated to 32 frames at a total dose rate of 60 e^−^/Å^2^ for each stack. Stacks were first motion corrected with MotionCorr and then binned 2-fold to a pixel size of 1.04 Å.

### Image processing

A simplified flowchart for the image processing procedure is presented in S3 Fig. We used GCTF for estimation of the contrast transfer function parameters and RELION 3.0 for all other image processing steps. Templates for reference-based particle picking were obtained from 2D class averages that were calculated from a manually picked subset of the micrographs. A total of 291,366 particles were automatically selected from a total of 1,916 micrographs. After 2D classification, 182,103 particles were selected and subjected to 3D classification with global angular searches of one class and 50 iterations. An initial model was generated with images of selected 2D class averages using RELION 3.0. The result of the last iteration was subjected to 3D classification with local angular searches of three classes and an angular step of 3.75° with C3 symmetry. A total of 99,489 good particles were selected from the 3D classification. These particles were then subjected to 3D autorefinement with local angular searches, resulting in a 3D reconstruction map with a resolution of 4.08 Å after postprocessing. A soft mask was generated with the resulting map and subjected to a second round of 3D autorefinement, resulting in a 3D reconstruction map with a resolution of 3.6 Å after postprocessing. The resolution was estimated with the gold-standard FSC 0.143 criterion [40]. Local resolution was estimated by ResMap [41].

### Atomic modeling

Initially, a homology model of hCNT3 (199–615) was built with SWISS-MODEL (http://swissmodel.expasy.org) using the structure of CNTnw (PDB code: 5L26) as a template. The model was manually placed in the map, and the “jiggle fit” option in Coot [42] was used to adjust the position of hCNT3. Residues 101–198 were manually built in Coot according to the density and distribution of TMs that were acquired by UniProt (https://www.uniprot.org/). The full model was finally refined in Phenix [43] using the real space refinement protocol.

### ITC

The nucleoside binding of hCNT3 was measured with a Micro-Cal ITC200. In total, 10 mM or 20 mM uridine/cytidine/thymidine was dissolved in the same buffer as the protein for titration at 25°C. The titration of nucleosides into the sample buffer was defined as a control and deducted. The first injection was removed during data processing. The data were analyzed by the PEAQ-ITC analysis software (MicroCal) using the one-site binding model. A representative result and the mean of Kd were presented. All the experiments were repeated three times (S1 Data).

## Supporting information

S1 Fig Purification of full-length hCNT3 and CNT3ins. Representative size-exclusion chromatograms of full-length hCNT3 and CNT3ins in buffer containing 10 mM HEPES/KOH (pH 8.0), 500 mM NaCl, 100 mM KCl, and 0.02% DDM (Superose 6 10/300). The peak fractions were subjected to SDS-PAGE and stained with Coomassie blue. CNT, concentrative nucleoside transporter; DDM, n-dodecyl-β-D-maltoside; hCNT, human CNT.
(TIF)

S2 Fig Pyrimidine nucleoside binding affinity measured by ITC. (A) Measurement of uridine binding affinity to CNT3 in sodium-free buffer. (B) Measurement of cytidine binding affinity to CNT3. (C) Measurement of thymidine binding affinity to CNT3. The underlying data for this figure can be found in S1 Data. CNT, concentrative nucleoside transporter; ITC, isothermal titration calorimetry.
(TIF)

S3 Fig Characterization and single-particle analysis of hCNT3. (A) A representative size-exclusion chromatography chromatogram of hCNT3 in the buffer containing 0.1% digitonin. The peak fractions were applied to SDS-PAGE. (B) The original electron micrograph of CNT3ins (Δ1–69). The green circles highlight representative particles. Representative two-dimensional class averages of the CNT3ins (Δ1–69) particles. (C) Angular distribution of the particles used for the final reconstruction of the CNT3ins (Δ1–69). Each cylinder represents one view, and the height of the cylinder is proportional to the number of particles for that view. Two orientations of the CNT3ins (Δ1–69) are shown. (D) Resolution estimation of the EM structure. The overall resolution was calculated to be 3.6 Å. FSC curves of the refined model versus the overall map that it was refined against (black), of the model refined in the first of two independent maps used for the gold-standard FSC versus that same map (red), and of the model refined in the first of two independent maps versus the second independent map (green). The small difference between the red and green curves indicates that refinement of the atomic coordinates did not suffer from overfitting. (E) Color-coded resolution variations in the CNT3ins (Δ1–69) structure as estimated by ResMap. CNT, concentrative nucleoside transporter; hCNT, human CNT; FSC, Fourier Shell Correlation.
(TIF)

S4 Fig Representative density maps of the structural elements of hCNT3. The densities are shown as blue mesh. CNT, concentrative nucleoside transporter; hCNT, human CNT.
(TIF)

S5 Fig Flowchart for EM data processing. Details can be found in Methods. EM, electron microscopy.
(TIF)

S6 Fig Sequence alignment of hCNT1 (UniProt no. O00337), hCNT2 (UniProt no. O43868), hCNT3 (UniProt no. Q9HAS3), vcCNT (UniProt no. Q9KPL5), and CNTnw (UniProt no. G4CRQ5). Sequences were aligned with ClustalW. Invariant and highly conserved residues are shaded yellow and gray, respectively. Residues responsible for substrate binding and sodium binding are indicated by blue circles, purple circles (Na1), and cyan circles, respectively. hCNT3 shares sequence identities of 47%, 43%, 39%, 36%, and 24% with hCNT1, hCNT2, vcCNT, CNTnw, and NupC, respectively. CNT, concentrative nucleoside transporter; hCNT, human CNT; NupC, nucleoside-proton cotransporter.
(TIF)

S7 Fig ConSurf evolutionary conservation analysis of proteins in the CNT family. Analysis was carried out automatically. Sequence identity was defined between 30% and 90%. There were 150 proteins from UniProt used in the analysis. hCNT3 is shown as a ribbon and is colored by ConSurf evolutionary conservation analysis. CNT, concentrative nucleoside transporter; hCNT, human CNT.
(TIF)

S8 Fig Superposition of the domains of vcCNT and CNT3ins. The core domains, scaffold domains, and transport domains of vcCNT and CNT3ins were extracted and are colored white and green, respectively. (A) Superposition of the core domains. (B) Superposition of the scaffold domains. (C) Superposition of the transport domains. The RMSD was calculated by PyMOL. CNT, concentrative nucleoside transporter; RMSD, root-mean-square deviation.
(TIF)

S9 Fig Uridine binding affinity of hCNT3 variants measured by ITC. Details can be found in the Methods. Each mutation of hCNT3 is labeled below the corresponding experimental result. The underlying data for this figure can be found in S1 Data. CNT, concentrative nucleoside transporter; hCNT, human CNT; ITC, isothermal titration calorimetry.
(TIF)

S10 Fig Uridine binding affinity of hCNT3 measured by ITC in low-pH conditions. The hCNT3 proteins were purified in 10 mM MES/KOH (pH 5.5), 500 mM NaCl, 100 mM KCl, 0.02% DDM and 10 mM MES/KOH (pH 5.5), 100 mM KCl, 500 mM choline chloride, 0.02% DDM, respectively. The underlying data for this figure can be found in S1 Data. CNT, concentrative nucleoside transporter; DDM, n-dodecyl-β-D-maltoside; hCNT, human CNT; ITC, isothermal titration calorimetry.
(TIF)

S1 Table Data collection, refinement, and model statistics. (XLSX)

S1 Data The summary of ITC results. ITC, isothermal titration calorimetry.
(XLSX)

## Abbreviations

2D: two-dimensional
3D: three-dimensional
CNT: concentrative nucleoside transporter
cryo-EM: cryogenic electron microscopy
DDM: n-dodecyl-β-D-maltoside
ENT: equilibrative nucleoside transporter
hCNT: human CNT
HP: hairpin-shaped helix
IH: interfacial helix
ITC: isothermal titration calorimetry
Kd: dissociation constant
LMNG: lauryl maltose neopentyl glycol
MelB: melibiose permease
PDB: Protein Data Bank
RMSD: root-mean-square deviation
SLC: solute carrier
TM: transmembrane helix
