# Supplementary figures and images for: Cryo-EM structure of the human concentrative nucleoside transporter CNT3
Source: PLoS Biol. 2020 Aug 10;18(8):e3000790. doi: 10.1371/journal.pbio.3000790 (PMC7440666; doi:10.1371/journal.pbio.3000790)

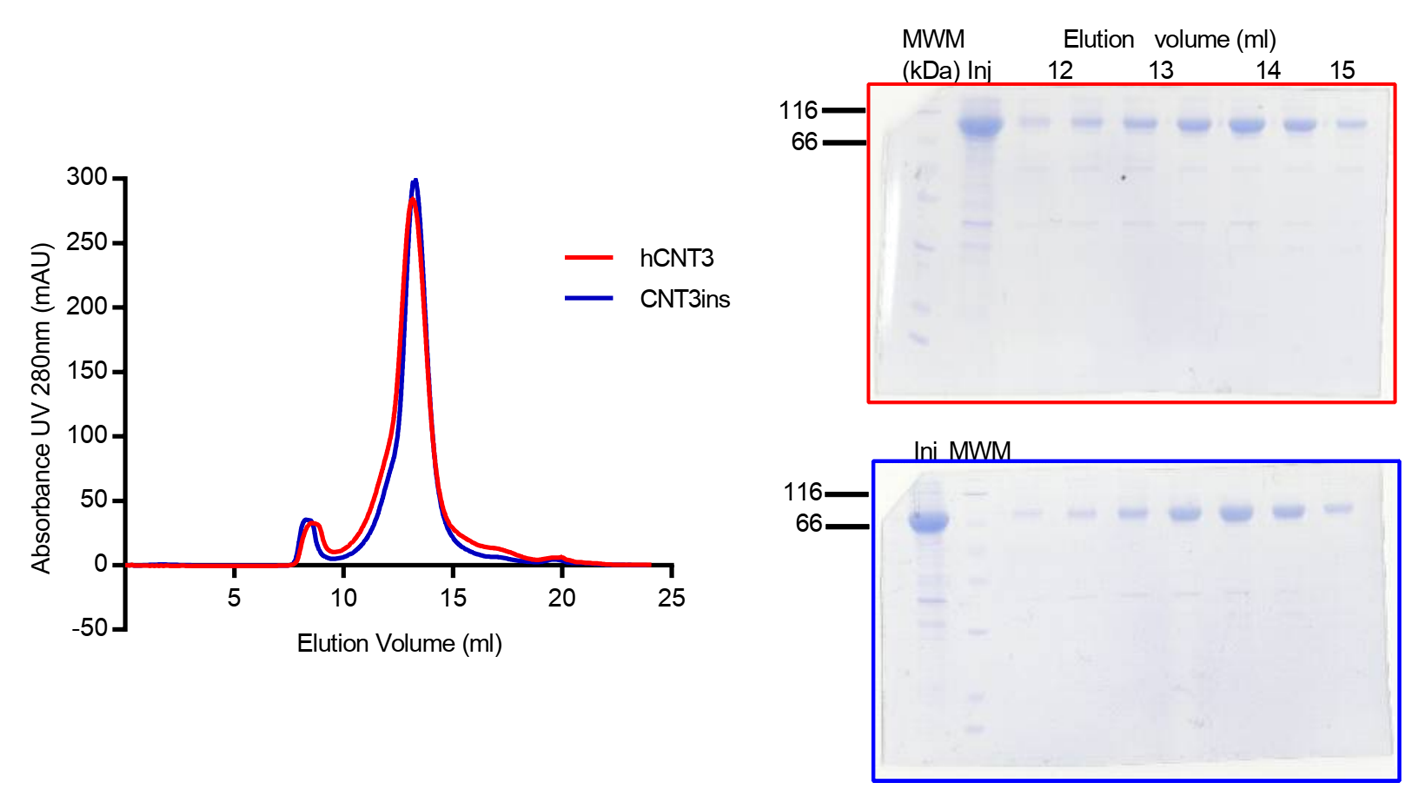

Supplement: S1 Fig — Representative size-exclusion chromatograms of full-length hCNT3 and CNT3ins in buffer containing 10 mM HEPES/KOH (pH 8.0), 500 mM NaCl, 100 mM KCl, and 0.02% DDM (Superose 6 10/300). The peak fractions were subjected to SDS-PAGE and stained with Coomassie blue. CNT, concentrative nucleoside transporter; DDM, n-dodecyl-β-D-maltoside; hCNT, human CNT. (TIF) [file pbio.3000790.s001.tif]

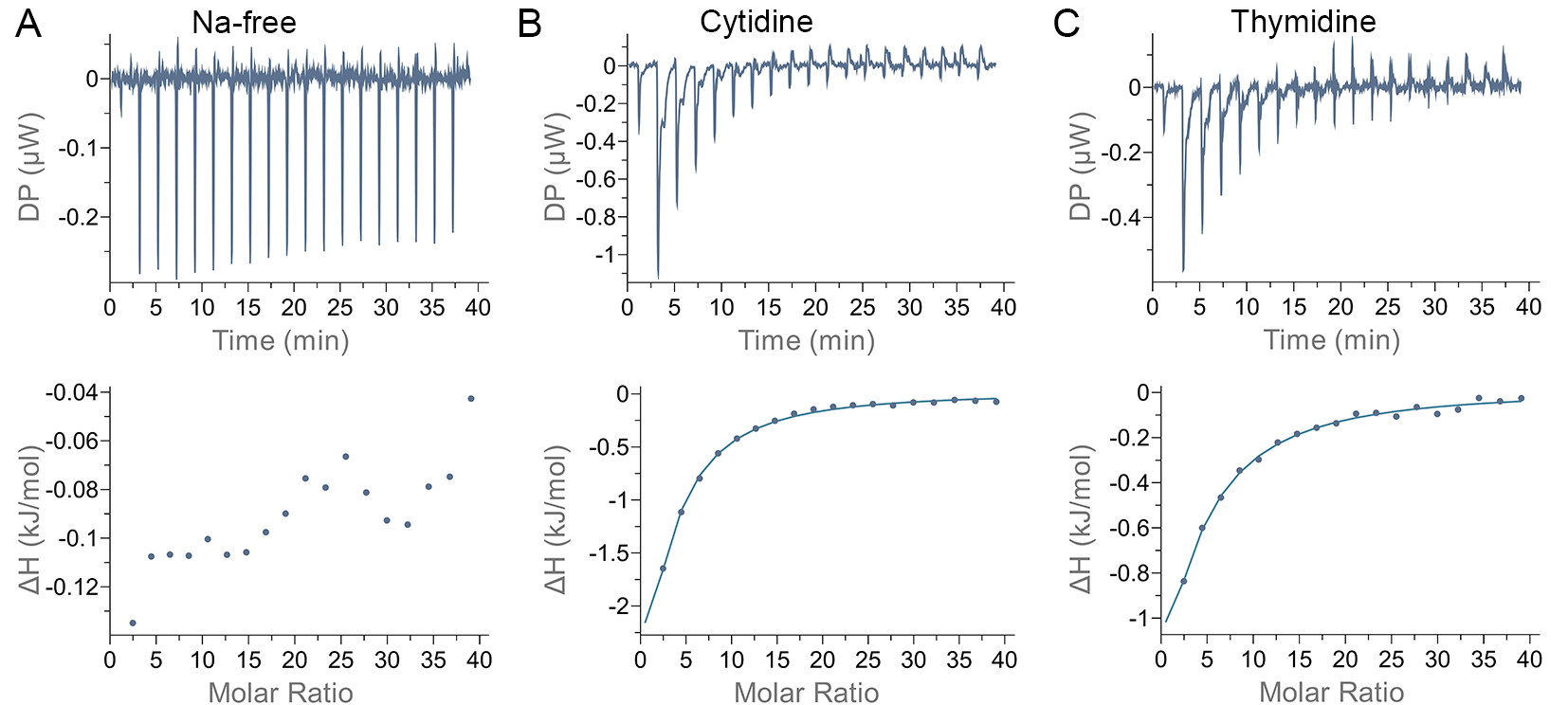

Supplement: S2 Fig — (A) Measurement of uridine binding affinity to CNT3 in sodium-free buffer. (B) Measurement of cytidine binding affinity to CNT3. (C) Measurement of thymidine binding affinity to CNT3. The underlying data for this figure can be found in S1 Data. CNT, concentrative nucleoside transporter; ITC, isothermal titration calorimetry. (TIF) [file pbio.3000790.s002.tif]

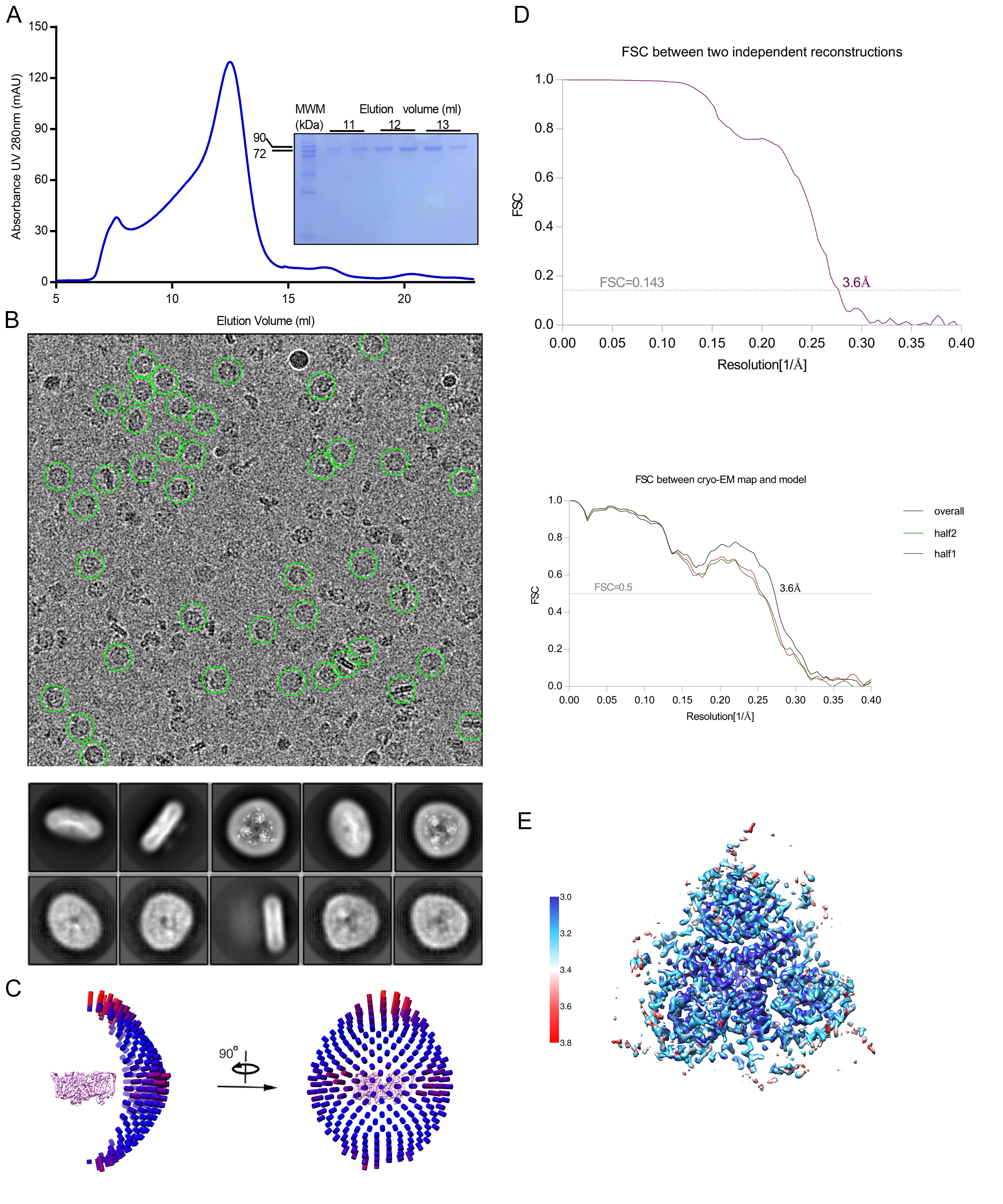

Supplement: S3 Fig — (A) A representative size-exclusion chromatography chromatogram of hCNT3 in the buffer containing 0.1% digitonin. The peak fractions were applied to SDS-PAGE. (B) The original electron micrograph of CNT3ins (Δ1–69). The green circles highlight representative particles. Representative two-dimensional class averages of the CNT3ins (Δ1–69) particles. (C) Angular distribution of the particles used for the final reconstruction of the CNT3ins (Δ1–69). Each cylinder represents one view, and the height of the cylinder is proportional to the number of particles for that view. Two orientations of the CNT3ins (Δ1–69) are shown. (D) Resolution estimation of the EM structure. The overall resolution was calculated to be 3.6 Å. FSC curves of the refined model versus the overall map that it was refined against (black), of the model refined in the first of two independent maps used for the gold-standard FSC versus that same map (red), and of the model refined in the first of two independent maps versus the second independent map (green). The small difference between the red and green curves indicates that refinement of the atomic coordinates did not suffer from overfitting. (E) Color-coded resolution variations in the CNT3ins (Δ1–69) structure as estimated by ResMap. CNT, concentrative nucleoside transporter; hCNT, human CNT; FSC, Fourier Shell Correlation. (TIF) [file pbio.3000790.s003.tif]

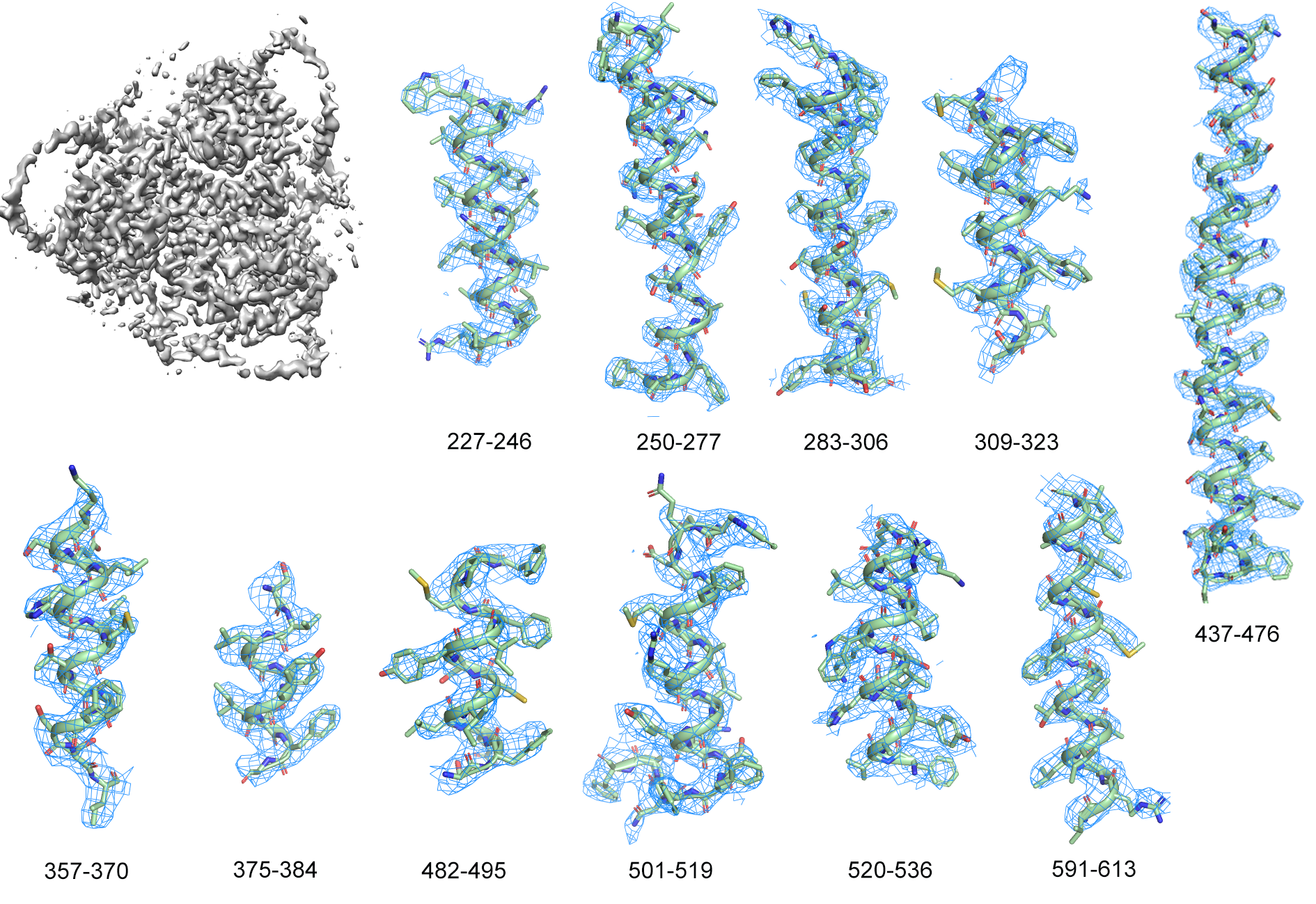

Supplement: S4 Fig — The densities are shown as blue mesh. CNT, concentrative nucleoside transporter; hCNT, human CNT. (TIF) [file pbio.3000790.s004.tif]

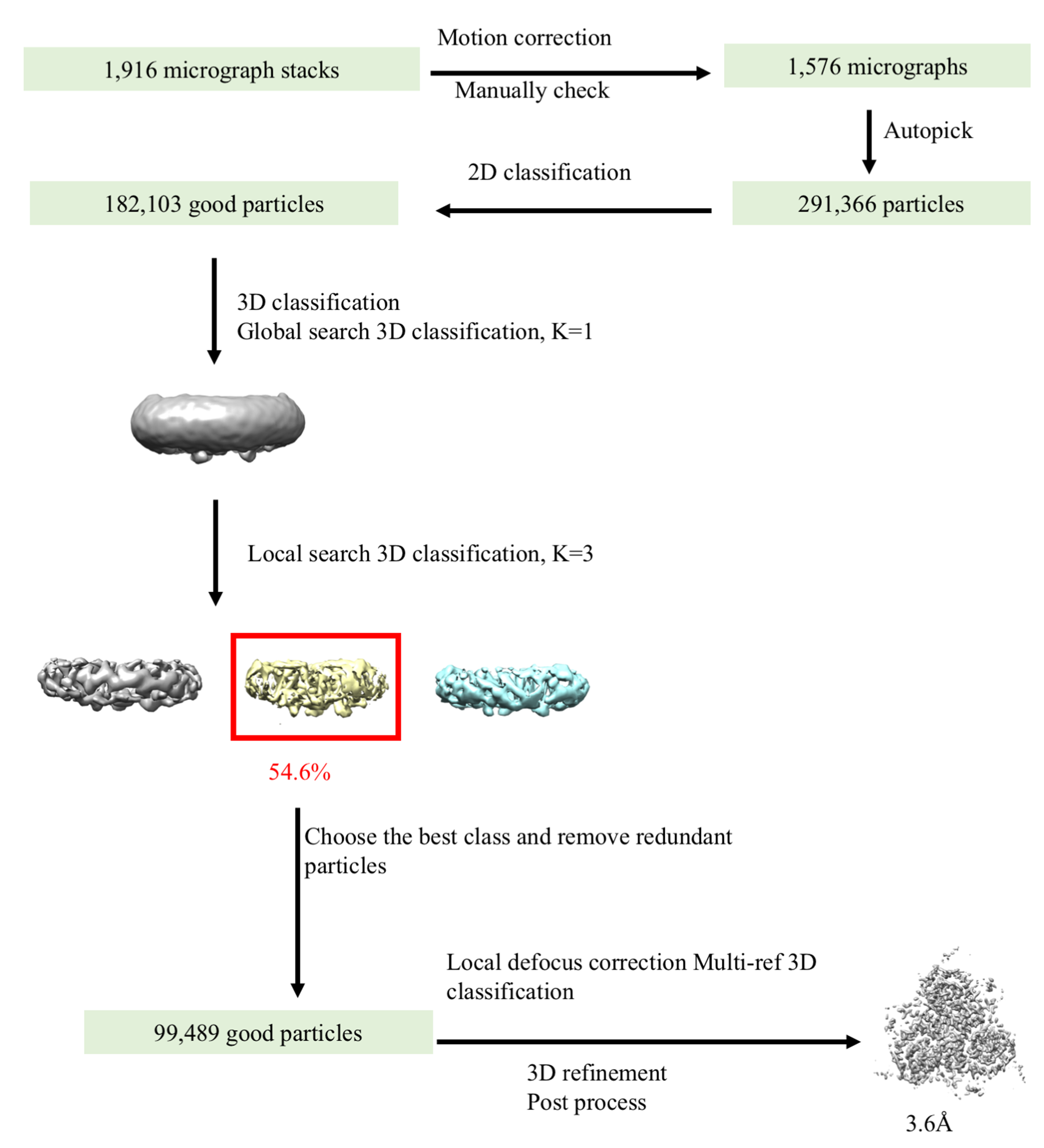

Supplement: S5 Fig — Details can be found in Methods. EM, electron microscopy. (TIF) [file pbio.3000790.s005.tif]

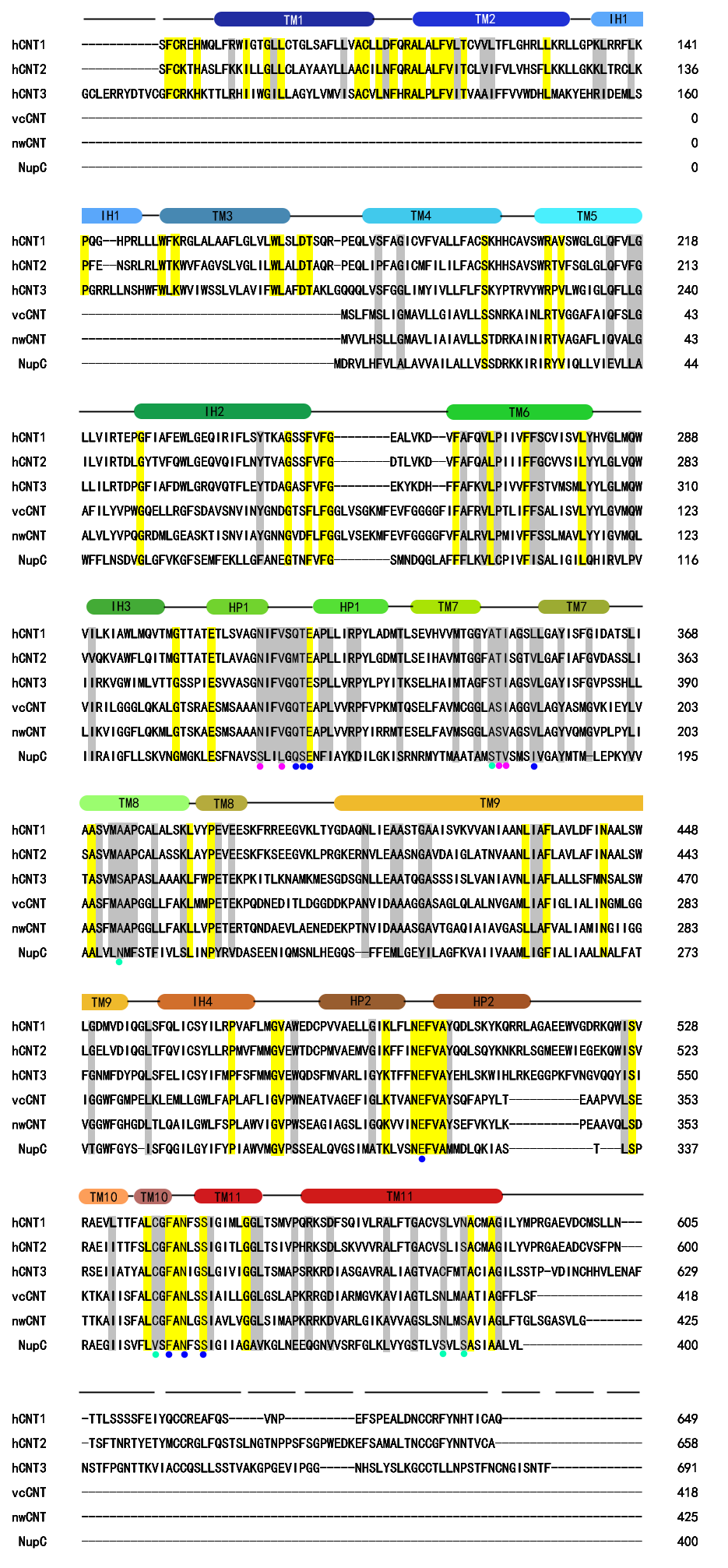

Supplement: S6 Fig — Sequences were aligned with ClustalW. Invariant and highly conserved residues are shaded yellow and gray, respectively. Residues responsible for substrate binding and sodium binding are indicated by blue circles, purple circles (Na1), and cyan circles, respectively. hCNT3 shares sequence identities of 47%, 43%, 39%, 36%, and 24% with hCNT1, hCNT2, vcCNT, CNTnw, and NupC, respectively. CNT, concentrative nucleoside transporter; hCNT, human CNT; NupC, nucleoside-proton cotransporter. (TIF) [file pbio.3000790.s006.tif]

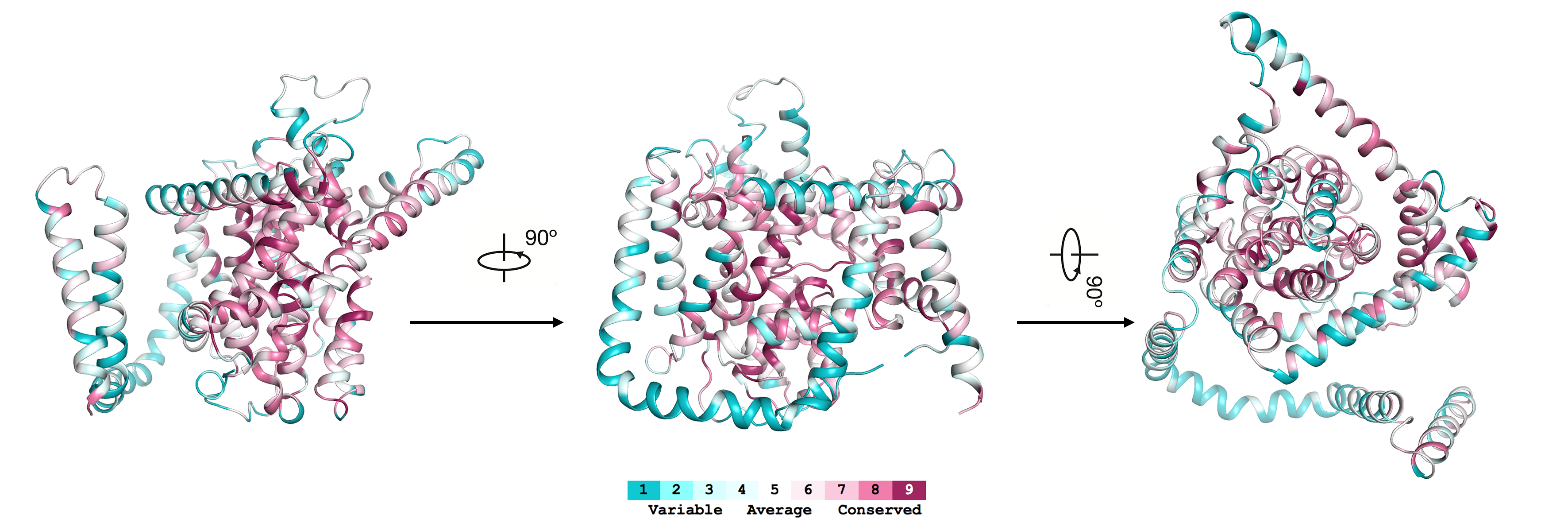

Supplement: S7 Fig — Analysis was carried out automatically. Sequence identity was defined between 30% and 90%. There were 150 proteins from UniProt used in the analysis. hCNT3 is shown as a ribbon and is colored by ConSurf evolutionary conservation analysis. CNT, concentrative nucleoside transporter; hCNT, human CNT. (TIF) [file pbio.3000790.s007.tif]

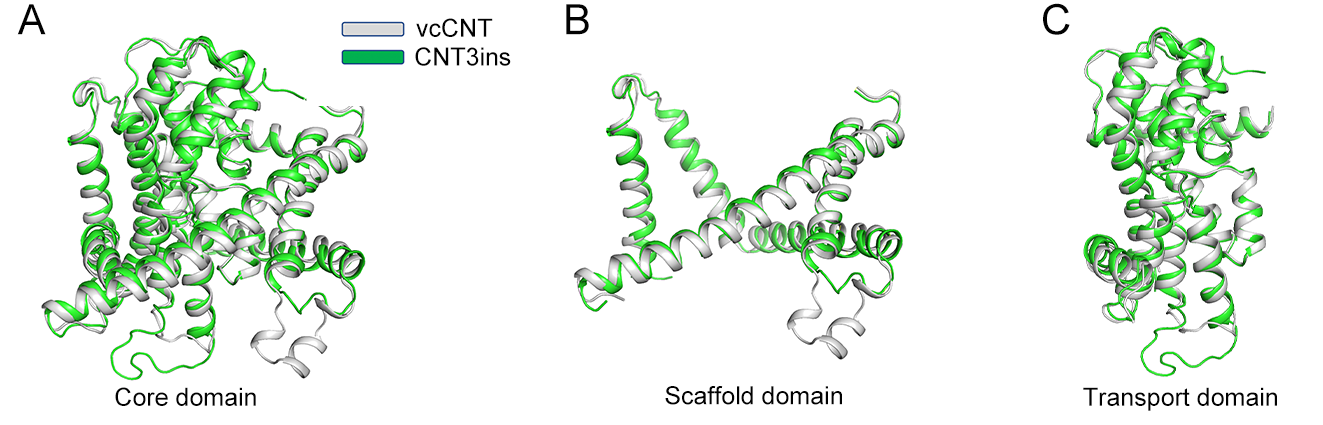

Supplement: S8 Fig — The core domains, scaffold domains, and transport domains of vcCNT and CNT3ins were extracted and are colored white and green, respectively. (A) Superposition of the core domains. (B) Superposition of the scaffold domains. (C) Superposition of the transport domains. The RMSD was calculated by PyMOL. CNT, concentrative nucleoside transporter; RMSD, root-mean-square deviation. (TIF) [file pbio.3000790.s008.tif]

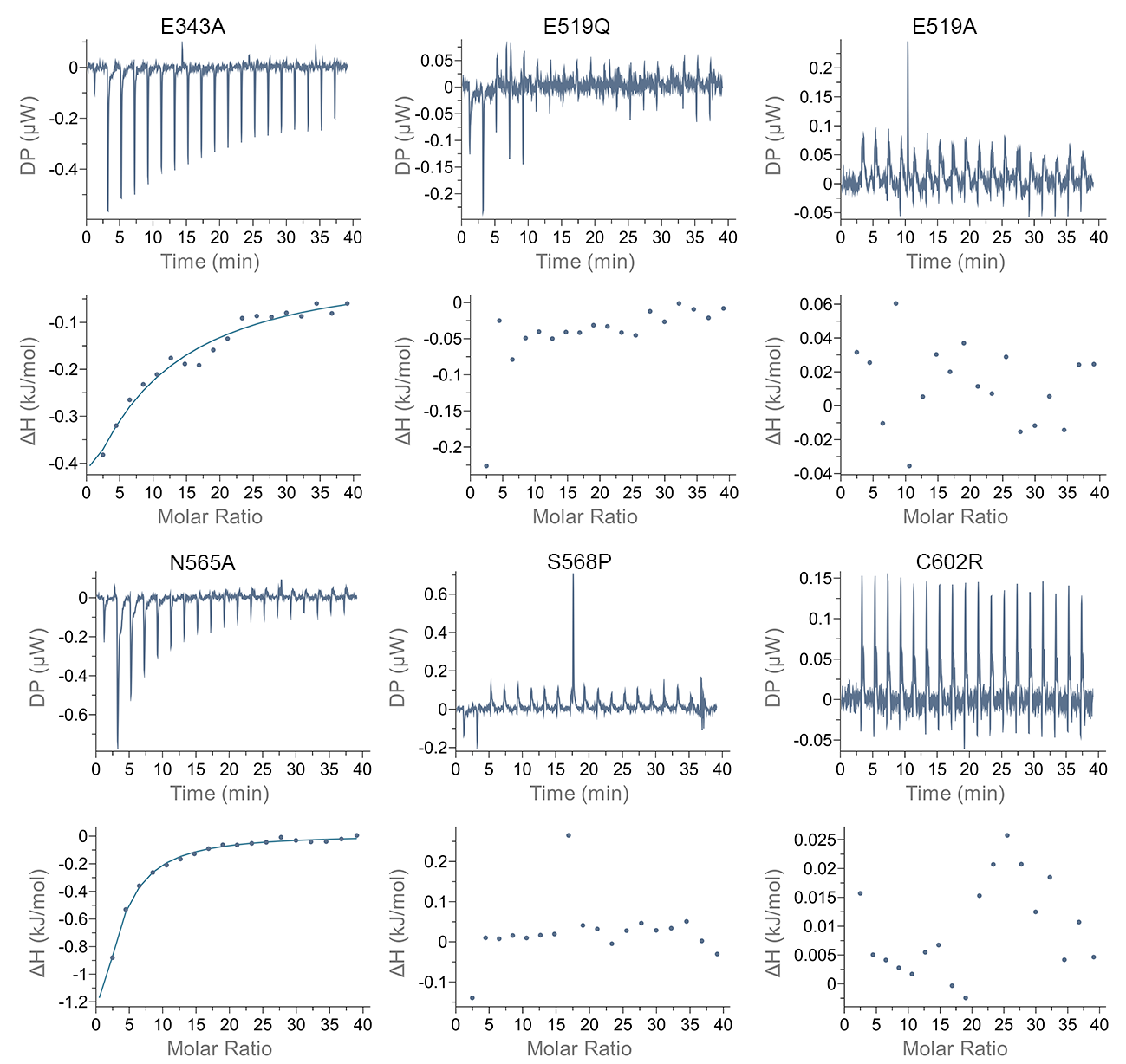

Supplement: S9 Fig — Details can be found in the Methods. Each mutation of hCNT3 is labeled below the corresponding experimental result. The underlying data for this figure can be found in S1 Data. CNT, concentrative nucleoside transporter; hCNT, human CNT; ITC, isothermal titration calorimetry. (TIF) [file pbio.3000790.s009.tif]

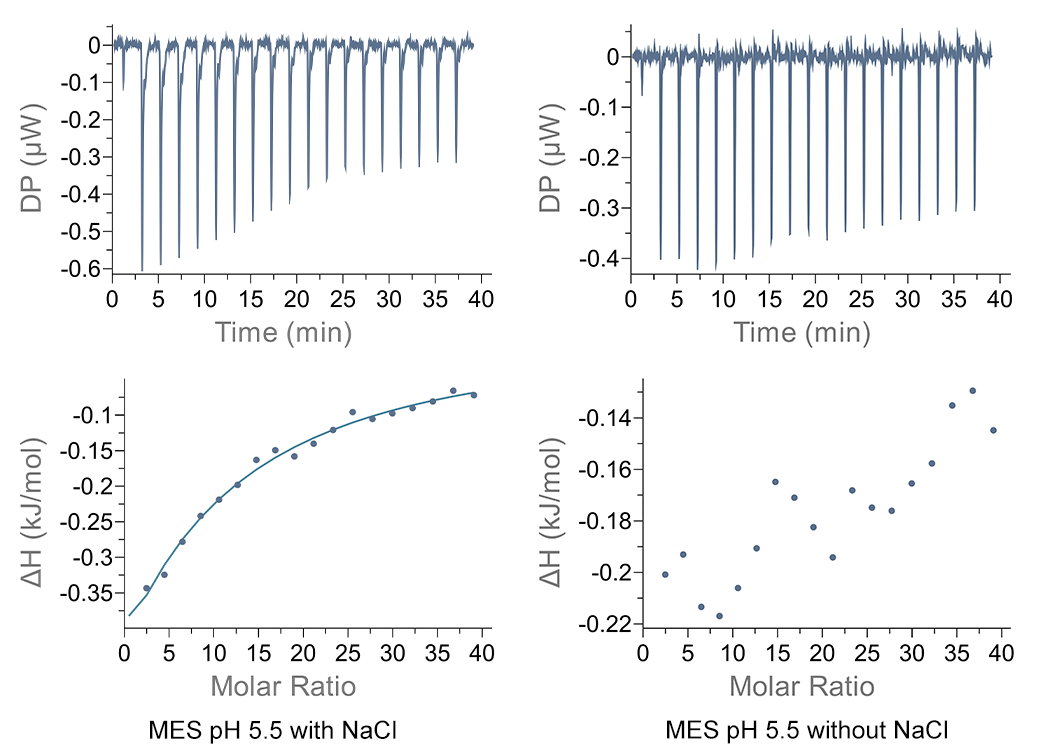

Supplement: S10 Fig — The hCNT3 proteins were purified in 10 mM MES/KOH (pH 5.5), 500 mM NaCl, 100 mM KCl, 0.02% DDM and 10 mM MES/KOH (pH 5.5), 100 mM KCl, 500 mM choline chloride, 0.02% DDM, respectively. The underlying data for this figure can be found in S1 Data. CNT, concentrative nucleoside transporter; DDM, n-dodecyl-β-D-maltoside; hCNT, human CNT; ITC, isothermal titration calorimetry. (TIF) [file pbio.3000790.s010.tif]
